# Probabilistic modeling to estimate jellyfish ecophysiological properties and size distributions

**DOI:** 10.1038/s41598-020-62357-5

**Published:** 2020-04-08

**Authors:** Simon Ramondenc, Damien Eveillard, Lionel Guidi, Fabien Lombard, Benoît Delahaye

**Affiliations:** 1 Sorbonne Université, CNRS, Laboratoire d’Océanographie de Villefranche, LOV, F-06230 Villefranche-sur-Mer, France; 2 Université de Nantes, CNRS, LS2N, F-44322 Nantes, France; 3 University of Hawaii, Department of Oceanography, Honolulu, HI 96822 USA

**Keywords:** Ecological modelling, Ecophysiology

## Abstract

While Ocean modeling has made significant advances over the last decade, its complex biological component is still oversimplified. In particular, modeling organisms in the ocean system must integrate parameters to fit both physiological and ecological behaviors that are together very difficult to determine. Such difficulty occurs for modeling *Pelagia noctiluca*. This jellyfish has a high abundance in the Mediterranean Sea and could contribute to several biogeochemical processes. However, gelatinous zooplanktons remain poorly represented in biogeochemical models because uncertainties about their ecophysiology limit our understanding of their potential role and impact. To overcome this issue, we propose, for the first time, the use of the *Statistical Model Checking Engine* (SMCE), a probability-based computational framework that considers a set of parameters as a whole. Contrary to standard parameter inference techniques, SMCE identifies sets of parameters that fit both laboratory-culturing observations and *in situ* patterns while considering uncertainties. Doing so, we estimated the best parameter sets of the ecophysiological model that represents the jellyfish growth and degrowth in laboratory conditions as well as its size. Behind this application, SMCE remains a computational framework that supports the projection of a model with uncertainties in broader contexts such as biogeochemical processes to drive future studies.

## Introduction

The ocean acts as a buffer against global warming^1^. However, understanding all processes and fluxes that lead to carbon sequestration is very complex and acts at multiple scales, from genes to ecosystems *via* physiological processes^2,3^. In particular, the ecosystem community structure and composition, from large organisms to molecular descriptions, is essential when studying biogeochemical processes and their variability^2^. In that community, gelatinous zooplanktons are still poorly represented compared to other groups^4^ such as silicifiers, calcifiers, or crustacean. In particular, their role in biogeochemical processes is still debated mainly due to the lack of information on the fate of the gelatinous biomass, even-though several recent studies support their inclusion in ecosystem models^5–7^. While there is no evidence that their abundances are globally increasing^8^, one observes their impact on carbon fluxes^9–11^ and the structuration of the trophic food webs^12–14^. Modeling the ecosystem structure to better represent biogeochemical processes in the ocean is, therefore, of primary importance. However, due to scaling issues and lack of holistic information on the plankton community, marine ecosystems are still very simplified compared to their terrestrial counterparts^15^. Today, the new generation of Ocean Biogeochemical Models called Dynamic Green Ocean Models allows resolving the biological complexity of marine ecosystems better thanks to the inclusion of multiple plankton functional types^15^. Similarly, trait-based modeling uses functional traits such as body size, shape, with a particular emphasis on trade-off to represent the ecosystem functioning^16,17^. Overall, general plankton compartments such as macrozooplankton or microphytoplankton summarize ecosystems in order to facilitate the parameterization of non-linear parameters. Nonetheless, such parameters remain difficult to determine because of their multi-scale implication, from physiology to ecosystems.

One of the main challenges in ecological modeling consists of acknowledging the whole biological complexity while remaining computationally tractable. To handle both opposite constraints, one advocates that a formal selection of the modeled species is a reliable solution^18^. The sensitivity of a model to one parameter might depend on the value of other parameters. In this setting, analyzing the sensitivity of the model to single parameters in isolation is not satisfying. Moreover, standard sensitivity analyses do not consider the number of simulations that are needed to obtain accurate results^19^. Following previous applications in engineering, the use of the Statistical Model Checking Engine (SMCE) overcomes this weakness by bringing formal confidence (trust) in the results while enhancing the range of sensitivity analysis towards considering global dependencies between parameters. First, instead of fixing parameter values to their mean observed values and performing sensitivity analysis of one parameter at a time, SMCE embeds uncertainty on parameter values inside the proposed models by assigning a probabilistic distribution to each parameter value (*i.e*., uniform distribution per default). SMCE performs model simulations by picking parameter values within their attached distributions (*i.e*., by considering the variances of parameters) and executing standard simulations. Thus, following several simulations, which implies considering several and distinct parameter choices, the SMCE performs a generalization of standard sensitivity analyses, not by analyzing the sensitivity of a single average simulation but rather by analyzing all feasible simulations and proposing general statistics of the whole; *i.e*., accurate statistical guarantees to perform predictive simulations while taking into account experimental uncertainties^20^. Overall, considering a predictive goal at both physiological and ecosystem levels, the SMCE produces a global set of parameter values that guarantees that the model matches experimental observations despite slight parameter variations.

In the Mediterranean Sea, *Pelagia noctiluca* is the most abundant scyphozoan species^21^. This holoplanktonic species is present all year long and has been observed without interruption since 1994^22^. Mostly present offshore, *P. noctiluca* reaches coastal waters thanks to wind events or sea currents variations^23,24^. Moreover, this species can realize nycthemeral migrations between 0 and 300–500 m deep. The development of the jellyfish *P. noctiluca* from oocyte to adult is strongly related to temperature and environmental food conditions^25–30^. It is a non-specific predator^21^ that responds rapidly to changes in the biotic and abiotic environment^31,32^. Indeed, like most of the scyphozoan species, *P. noctiluca* has been known to shrink its body mass when prey concentration becomes limiting^33–37^.

Because *P. noctiluca* is a multi-scale player (*i.e*., strong interaction from microbial communities to high trophic level), estimating its model parameters is difficult. The goal of this paper is to apply the SMCE to infer the parameters of an ecophysiological model and discuss the putative importance of a jellyfish in marine ecosystems and biogeochemical processes in the Mediterranean Sea. According to our knowledge, the SMCE approach was never used before in this context. To this purpose, we (*i*) build an ecophysiological model for *P. noctiluca* to describe the fundamental physiological processes that are involved in carbon fluxes, (*ii*) infer the model’s parameters using the SMCE and (*iii*) discuss the potential contribution of *P. noctiluca* egestion to POC fluxes despite missing knowledge. The benefits of the SMCE consist not only in performing an accurate parameter estimation on several scales (from laboratory physiological experiments to *in situ* biomass distribution) simultaneously but also in emphasizing correlations between parameters of its ecophysiological model through automatic analysis. Here *P. noctiluca* is used as a scaling-up example of how one can apply state of the art verification methods in computer sciences to better estimate parameters of an ecophysiological model.

## Results

### Conceptual model

**Figure 1:**
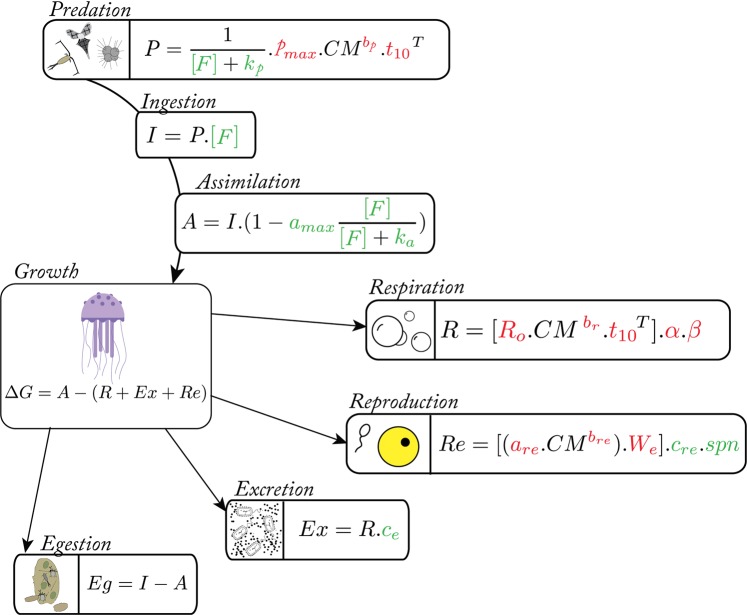
Conceptual diagram and equations used in the *Pelagia noctiluca* ecophysiological model. Arrows between compartments represent the biological carbon transfer following the ecophysiological processes. Symbols and units of the different variables are described in Table. S1. The red and green colors represent all parameters deduced from the literature dataset and Statistical Model Checking Engine (*SMCE*) respectively.

To reproduce the jellyfish growth and degrowth in captivity and wild conditions, an ecophysiological model was built based on seven physiological processes (predation, ingestion, assimilation, respiration, excretion, reproduction, egestion) and constrained by 17 parameters (see Fig. 1 for illustration and methods for details). Eleven parameters (*p*_*max*_, *b*_*p*_, *t*_10*p*_, *R*_*o*_, *b*_*r*_, *t*_10*r*_, *α*, *β*, *a*_*re*_, *b*_*re*_, *W*_*e*_; Table 1) were defined by previous experimental data whereas six unknown parameters (*k*_*p*_, *a*_*max*_, *k*_*a*_, *c*_*re*_, *spn*, *c*_*e*_; Table 1) were inferred using SMCE. During the simulations, the jellyfish carbon mass (CM) prediction was constrained by two forcing variables: temperature and zooplanktonic biomass. In controlled conditions, the temperature was fixed at 18 °C following Lilley, *et al*.^38^ whereas prey concentration was null or estimated from a prey concentration range (F_lab_; Table 1) in degrowth and growth experiments, respectively. In the *in situ* conditions, these variables were obtained by an annual climatology of sea surface temperature and zooplankton concentration from 2011 to 2015 in the bay of Villefranche-sur-Mer, France.

**Table 1:** Values, symbols, description and units of the different parameters used in the model.

Ecophysiological process	Parameters	Values	Units	Description
*Predation (P)*	*p*_*max*_	0.1399	*gC.ind*^*−1*^*.d*^*-1*^	Theoretical predation rate of 1 g CM individual at 0 °C and with unlimited food; log(C) = b.log(B) + a (adapted from Acuna, *et al*.^66^)
	*b*_*p*_	0.8856	dimensionless	
	*k*_*p*_	[3 × 10^*−*5^: 2 × 10^*−*5^: 2 × 10^*−*4^]	*gC.L*^*−1*^	Half saturation coefficient for predation (this study)
*Respiration* (*R*)	*R*_*o*_	2.80	*μmolO*_2_.d^*−*1^	Theoretical respiration rate of 1 g WM individual at 0 °C (adapted from Lilley, *et al*.^34^)
	*b*_*r*_	0.934	dimensionless	Allometric exponent for the effect of individual mass on respiration rate (adapted from Lilley, *et al*.^34^)
*Reproduction* (*Re*)	*a*_*re*_	0.07	dimensionless	Allometric exponent for the production of eggs (adapted from Lilley, *et al*.^34^)
	*b*_*re*_	4.66	dimensionless	
	*W*_*e*_	1.52 × 10^*−*6^	*gC.eggs*^*−*1^	Eggs carbon mass^34^
	*c*_*re*_	[1: 0.1: 3]	dimensionless	Proportion of mucus production during spawn (this study)
	*spn*	[0.4: 0.1: 1]	*d*^*−*1^	Spawning rate (this study)
*Assimilation* (*A*)	*a*_*max*_	[0.5: 0.1: 1]	dimensionless	Maximal assimilation rate (this study)
	*k*_*a*_	[1.5 × 10^*−*5^: 0.5 × 10^*−*5^: 5 × 10^*−*5^]	*gC.L*^*−1*^	Half saturation coefficient for assimilation (this study)
*Excretion (Ex)*	*c*_*e*_	[0: 0.2: 2]	dimensionless	Proportion of excretion production (this study)
*Conversion factor*	*β*	447 × 10^*−*3^	*J.μmolO*_2_^*−*1^	Coefficient to convert from oxygen units to energy units^67,74^
	*α*	2.28 × 10^*−*5^	*gC.J*^*−*1^	Coefficient to convert from energy units to carbon units^67^
	*t*_10_	1.066	dimensionless	Tenth root of the Q_10_ coefficient which describes by how much as a rate changes with a 10 °C increase in temperature (adapted from Lilley, *et al*.^34^)
*Food concentration*	*F*_*lab*_	[0.7 × 10^*−*5^: 0.1 × 10^*−*5^: 1.2 × 10^*−*5^]	*gC.L*^*−1*^	Prey concentration in laboratory condition (this study)

### Implementation of the statistical model checking engine

**Figure 2:**
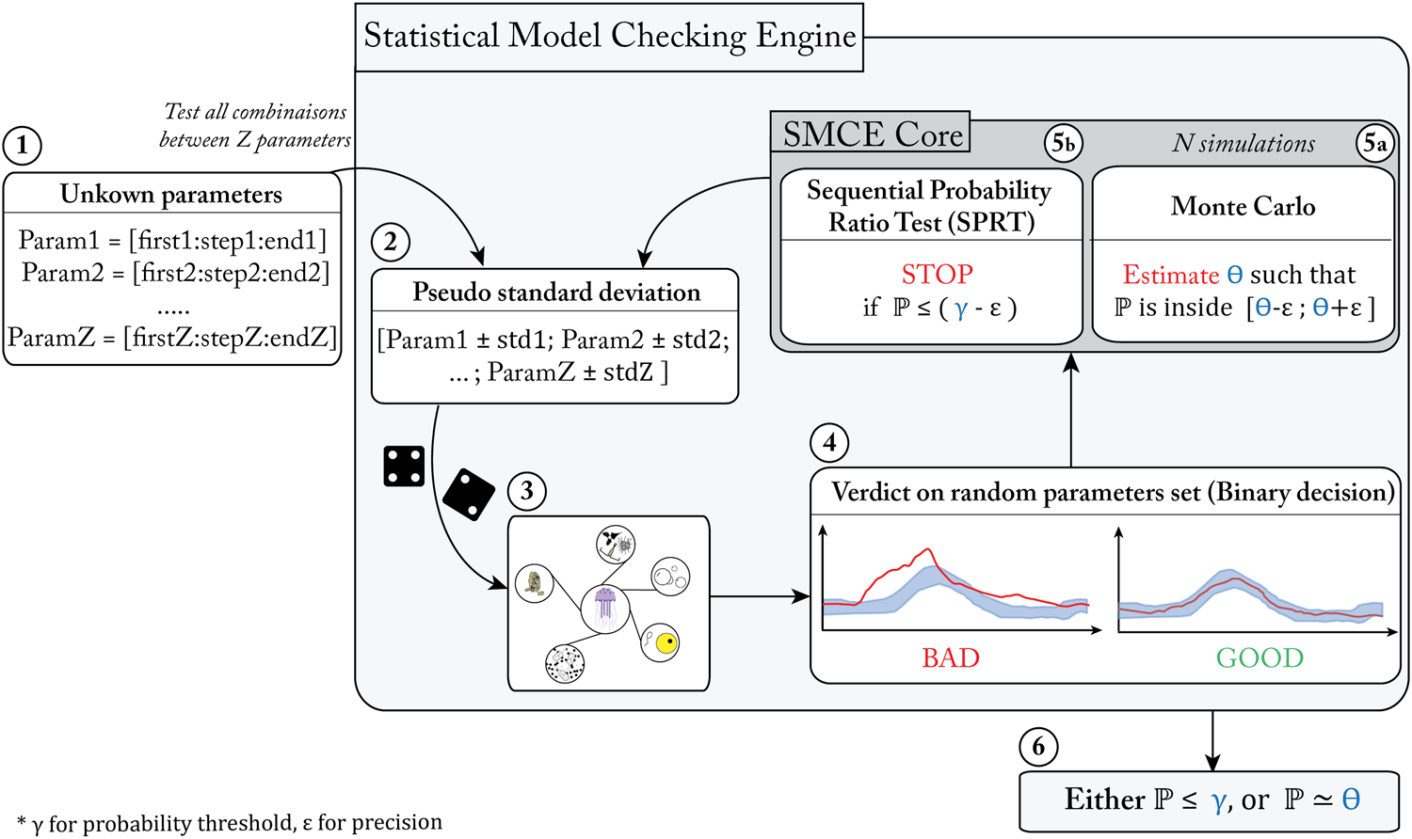
The activity diagram of the *SMCE* used to find the best combination of values for the unknown parameters in the ecophysiological model.

The SMCE is a new mathematical approach based on probability (${\mathbb{P}}$). The core of this method consists of a combination of the Monte Carlo method^39^ and the Sequential Probability Ratio Test (SPRT)^40^. Implemented within a computational framework, the SMCE allows simulating models or programs with uncertainties (Fig. 2). In particular, the SMCE allows a modeler to search for the optimal parameter values concerning experimental data. Initially, the modeler assigns a virtual search vector, with upper bounds, lower bounds, and resolution chosen according to prior knowledge, to each unknown parameter (step.1 in Fig. 2; Table 1). This vector allows defining a global search domain for optimal parameter values as the Cartesian product of the individual search vectors of all unknown parameters. Consequently, the size of the search space, as well as the computing time, increases proportionally following the width and the resolution of the individual vectors of each unknown parameter (*i.e*., the size range of values subjects to investigation). In addition, instead of considering exact parameter values for each sample in the search space, SMCE will take into account uncertainty by using standard deviations around a sample before measuring its adequation to experimental datasets. Briefly, the essence of the SMCE is as follows: for each set of parameter values *Param* chosen from the sample space, a pseudo standard deviation (*std*) is used to create a new interval [*Param* – *std*: *Param* + *std*] (step.2 in Fig. 2), yielding a probabilistic model (*i.e*., a model where the parameter values are chosen according to a uniform distribution on their intervals). The size of this deviation is chosen according to prior knowledge of the variability of each unknown parameter.

With such a probabilistic model, one can define a probability measure on the outputs of its simulations. Ideally, the score obtained by SMCE should reflect the probability measure of the set of outputs that best match experimental datasets. Unfortunately, computing the exact measure (written ${\mathbb{P}}$) is very costly. Therefore, the SMCE resorts to statistical analysis in order to compute an estimation of ${\mathbb{P}}$. In order to do so, the SMCE performs many simulations of the model according to the chosen parameter set. At each simulation, random values of the parameters are chosen from their interval, which takes into account variability, and implemented in the model before running it (step.3 in Fig. 2). Model outputs are then compared to observations before running the SMCE to inform the latter whether the predictions were correct or not (*i.e*., *True* or *False*) (step.4 in Fig. 2). From those random simulations, the Monte Carlo technique allows computing a value Θ which is an estimation of the measure of ${\mathbb{P}}$, with a degree of precision ε that depends on the chosen number of simulations and represents the adequation of the obtained prediction concerning the given data (step.5a in Fig. 2). As a consequence, obtaining an estimation Θ of the measure ${\mathbb{P}}$ ensures that ${\mathbb{P}}$ lies in the interval [Θ − ε; Θ + ε]^41^.

In order to spare computing time, the Monte Carlo technique has been combined with the SPRT method. This approach allows stopping the estimation of ${\mathbb{P}}$ (hopefully before all the simulations have been performed) if the simulations that have already been performed ensure that this probability cannot be greater than a threshold (γ) (step.5b in Fig. 2). When all the necessary simulations have been performed, the SMCE either returns the estimation Θ of ${\mathbb{P}}$ if it is high enough or a statement that ${\mathbb{P}}$ is below the threshold otherwise. Such estimation is performed for each set of parameter values from the search space (step.6 in Fig. 2). The estimated measures of all sets of parameter values are finally compared, which allows identifying the set of parameter values that gives the best prediction for the experimental data.

To summarize, the SMCE estimates the probability ${\mathbb{P}}$ for each set of parameter values (before adding the pseudo *std*) thanks to the Monte Carlo or SPRT approach by comparing individual simulations of the model with our experimental data. One accomplished such a comparison *via* a “tunnel” selection based on the standard deviations of our experimental datasets. Considering a tunnel as the range of acceptable model outputs, a simulation of a model for a given set of parameter values is “correct” if the outputs of this simulation fit inside the tunnels (see step.4 in Fig. 2 for illustrations).

### Calibration of the ecophysiological model

**Figure 3:**
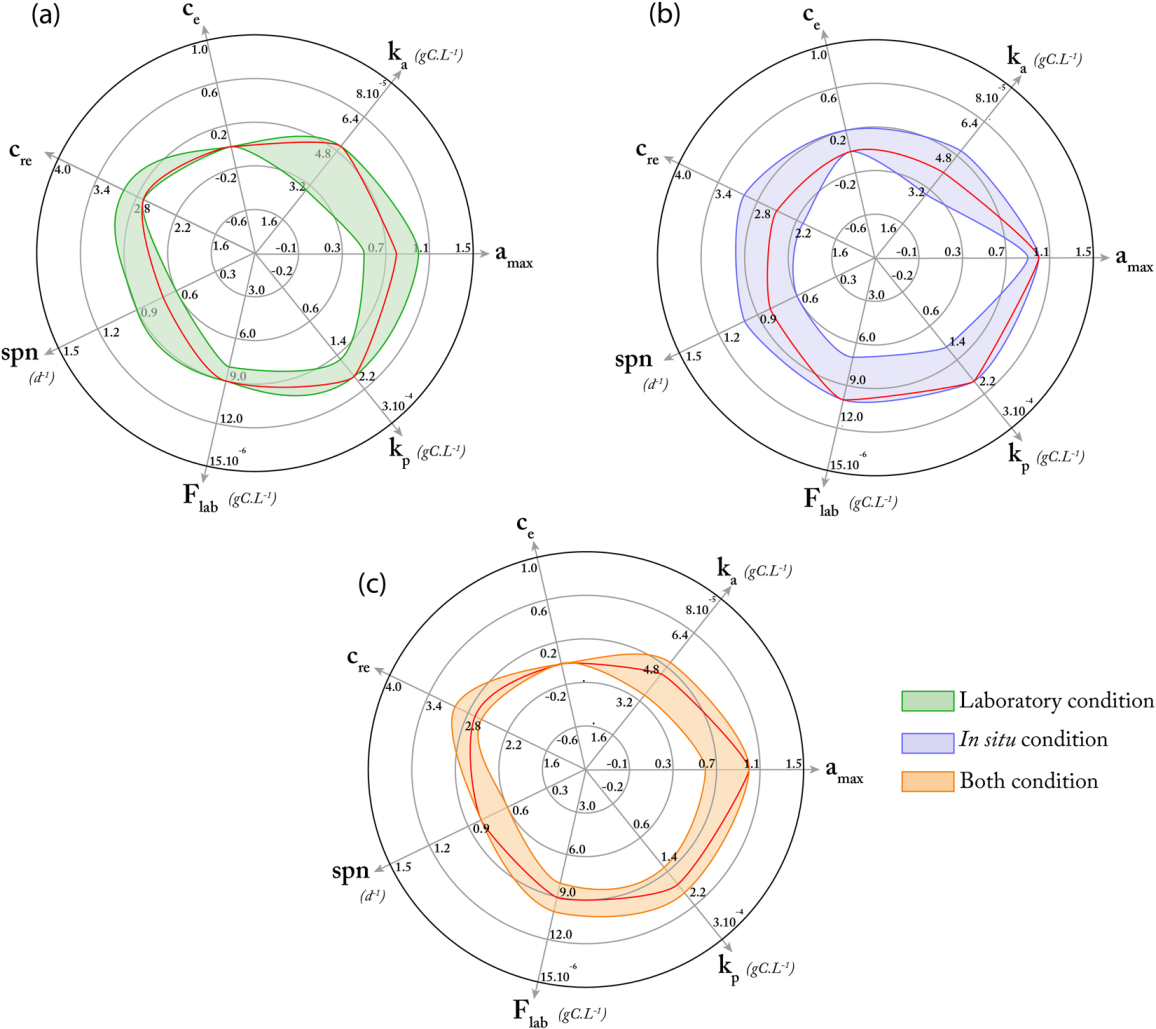
Vectors of the central points of the optimal parameter distributions for the growth rates measured in the laboratory (**a**), *in situ* conditions (**b**) and according to both (**c**). Color areas represent upper and lower bounds of the central points for the 50 best vectors of optimal parameter distributions. For each parameterization condition, the best vector is depicted by red lines.

The use of SMCE allows defining vectors of optimal parameter values (*i.e*., central values for the distributions of each parameter) within the parameter space. While originally designed as a deterministic model with a mechanistic description of the biological processes, SMCE provides an estimation of a set of vectors of optimal parameter values that transforms the model into a probabilistic one (*i.e*., parameter values are described as a distribution of values). The set of vectors could change if one considers the field and/or laboratory conditions (see the color-filled zones in Fig. 3 for illustration).

**Figure 4:**
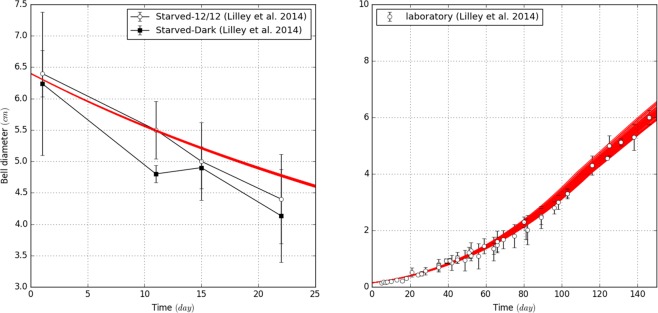
Comparison between the results of the laboratory experiments and the outputs of the ecophysiological model, based on fifty random simulations within the best parameters combinations defined by the *SMCE*. Subpanels show degrowth and growth simulation in (**a**) starved and (**b**) feeding conditions respectively.

The best parameters set selected in controlled condition corresponds to a spawning frequency of 0.7 days (*spn* = 0.7 ± 0.05), a maximum fraction of assimilated food of 80% (*a*_*max*_ = 0.8 ± 0.05), a half-saturation assimilation and predation constants of 5.10^−5^ (*k*_*a*_ = 5.10^−5^ ± 5.10^−6^) and 1.9.10^−4^ (*k*_*p*_ = 1.9.10^−4^ ± 1.10^−5^) gC.L^−1^ respectively, a very low excretion rate (*c*_*e*_ = 0 ± 0.025), a carbon cost for mucus production equal to 2.7 times the carbon-cost of eggs production (*c*_*re*_ = 2.7 ± 0.05) and a prey concentration around 9.10^−6^ gC.L^−1^ (Fig. 3a). The representation of 50 random simulations from the best parameter set highlights that the ecophysiological model correctly represents the jellyfish growth and degrowth in laboratory conditions (Fig. 4). In starved conditions, the model shows that the jellyfish bell diameter decreases exponentially due to carbon losses *via* respiration, excretion, and reproduction (Fig. 4a). At the beginning of the degrowth experiment, respiration, excretion, and reproduction are responsible respectively of 47%, <1% and 53% of the total organic carbon loss, compared to 62%, <1% and 38% at the end of simulations. However, the model slightly underestimates the biomass losses (~11% compared to the average of the measurements). In contrast, in feeding conditions (Fig. 4b), jellyfishes grow exponentially throughout the somatic phase, reaching saturation when sexually mature though prey concentration stays constant in the environment. This equilibrium suggests that the amount of organic carbon gained by predation is equal to losses promoted by excretion, egestion, and respiration. Thus, along with other marine species, *P. noctiluca* growth can be represented by a sigmoid curve.

**Figure 5:**
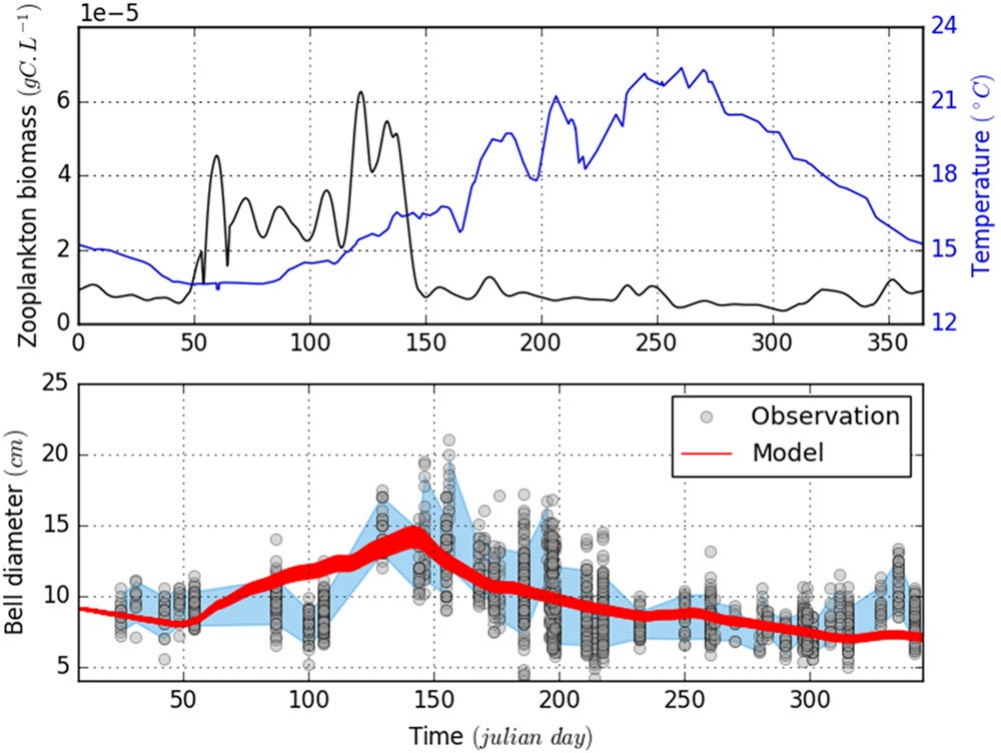
Comparison of jellyfish size variations between *in situ* observations and outputs of the model, based on fifty random simulations within the best parameters combinations defined by the *SMCE*. Subpanels show: (**a**) the annual sea surface temperature (blue) and annual zooplankton biomass (black) climatologies (from August 2011 to December 2015) that were used as model inputs; and (**b**) the jellyfish size variation obtained from *in situ* observations (dots) and model simulations (red lines). The blue color represents the tunnel of confidence for our *SMCE* decision.

In the *in situ* conditions, the 2011–2015 annual climatology of temperature and zooplankton biomass in the northwestern Mediterranean Sea (Fig. 5a) showed significant seasonal variations. In winter, both sea surface temperature (SST) and zooplankton biomass are low (13 °C, and 1.10^−6^ gC.L^−1^, respectively). In early spring, heating of the water column leads to a stratification allowing phytoplankton growth which, in turn, generates an increase of zooplanktonic biomass (5-6.10^−6^ gC.L^−1^). During summer, the zooplankton biomass decreases to 1.10^−6^ gC.L^−1^ while the SST increases to 22 °C, before decreasing below 18 °C in autumn. According to our observations (*n* = 1734), the size of *P. noctiluca* ranges between 2.1 and 21 cm throughout the year and is strongly related to environmental conditions. Three phases characterize *P. noctiluca* growth: (*i*) a slow growth in winter and early spring, (*ii*) an exponential growth as soon as the temperature and food availability increase in mid-spring, and (*iii*) a degrowth phase during summer and autumn associated to massive gametes emission^35,36,42,43^ and respiration, which is not compensated by feeding on the scarce food available.

Compared to laboratory conditions, the best parameter set selected corresponds to spawning frequency of 0.8 (*spn* = 0.8 ± 0.05), a maximum of assimilated food around 100% (*a*_*max*_ = 1.0 ± 0.05), a half-saturation constant assimilation and predation near 4.10^−5^ (*k*_*a*_ = 4.10^−5^ ± 5.10^−6^) and 1.9.10^−4^ (*k*_*p*_ = 1.9.10^−4^ ± 1.10^−5^) gC.L^−1^ respectively, an excretion rate close to 0% of the respiration rate (c_e_ = 0 ± 0.025) and mucus production equal to 2.5 time the carbon-cost of eggs production (*c*_*re*_ = 2.5 ± 0.05). The representation of 50 random simulations from the best parameter set shows a good agreement between adult size observations and simulations (Fig. 5b). However, an unusual event at 100^th^ Julian day could not be captured by the simulations and by any parameter sets. This mismatch occurs during the early spring season when observations of jellyfish are difficult because of sea state conditions. In addition, these abiotic perturbations can also impact the vertical distribution of the jellyfish leading to potentially biased observations. Interestingly, Milisenda *et al*.^44^ showed the occurrence of different cohorts in the Messina Strait throughout the year. Compared to the size measurements carried out in this study, all observations captured mainly the size distribution of the highest cohort (adult) except in April where the observations were similar to the intermediate cohort. This result suggests that simulations do not overestimate and reproduce correctly adult size distribution while the April observations could be biased by a younger cohort.

Finally, satisfying both laboratory and *in situ* conditions, we do not introduce more variability on each individual parameter but found a compromise for the best parameter set for both conditions (Fig. 3c). Here, the goal of SMCE is to identify a set of parameters that matches all observations (laboratory experiments and *in situ* observations). In this context, the errors are the sum of errors from laboratory and *in situ* conditions. Special attention has been given to the time resolution in order not to advantage of one condition compared to the other.

## Discussion

In the last few years, computer sciences promoted the use of the SMCE method for verifying large software models that are out of reach of standard verification methods. Its purpose is to analyze a software model in order to prove (or disprove) that it satisfies desirable properties. Although marginally applied to non-software systems (*e.g*., aeronautics^45^ or gene regulatory networks^46^), its use has always required extensive computational skills (*i.e*., use of dedicated modeling languages). Here we propose (*i*) to apply SMCE to biological models *per se* with no pervasive modification and (*ii*) to reach parameter estimation expectations while (*iii*) allowing a gain of global knowledge on the models besides the verification of predefined properties. While other efficient methods exist for parameter estimation^47^, such as MCMC^48^ or others that consider uncertainties^49^, SMCE brings several novel aspects that cannot be obtained through existing methods. In particular, SMCE, while being inherently probabilistic, can be applied to models originally designed as deterministic ones by using distributions on parameter values. While deterministic models tend to represent the behavior of an *average system*, their probabilistic version allows representing the whole community instead. Thus, probabilistic models take into account the inherent variability of parameter values that inevitably emerges when extracting a trait parameter value from experiments that, by essence, consider a community of individuals rather than a single individual. Aside from these experimental uncertainties, the use of probabilistic models also allows taking into account uncertainties that arise from incomplete knowledge such as those produced by incomplete mechanistic descriptions in the model, or ambiguities about initial conditions^50^.

**Figure 6:**
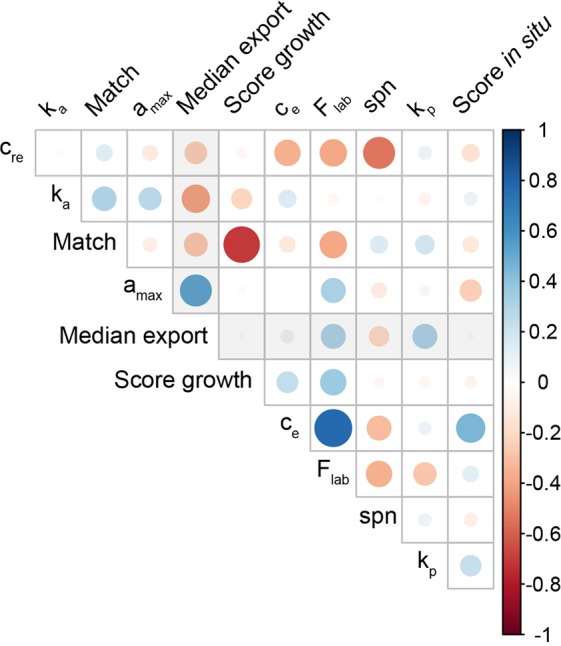
Correlogram representing Spearman’s correlation rank between input variable (F_lab_) and parameters (*k*_*p*_, *a*_*max*_, *k*_*a*_, *c*_*e*_, *c*_*re*_, *spn*), output variables obtained by the SMCE (Match, Score growth, Score *in situ*), and the contribution of POC exported due to jellyfish (Median export). Color scale and circle size indicate the strengths of the correlation.

While standard methods, such as MCMC parameterization techniques, are highly efficient, their purpose remains to find one vector of optimal values within the parameter space. Aside from the fact that the resulting parameterized models will remain mainly deterministic, these methods also do not benefit from an extensive analysis of the parameter space. On the contrary, SMCE uses such an analysis to bring additional insights. Indeed, SMCE provides an estimation of a set of vectors of optimal parameter values (again, central values for the distributions of each parameter) that could change if one considers *in situ* or laboratory conditions (see the color-filled zones in Fig. 3 for illustration). Each vector of optimal parameter values is certified, and Fig. 3 presents one of them in red for the sake of illustration. However, we advocate herein to consider the whole set of certified vectors to learn dependencies between parameters, as pictured in the correlogram Fig. 6, that are usually out of reach of standard sensitivity analysis. The benefits of this analysis are multiple. By embedding uncertainties with the models, one can (*i*) link parameters that concern different biological scales within the same model analysis, and (*ii*) identify independent parameters that are insights for reducing the complexity of models during their design.

For the sake of model validation, the best parameter values found by the SMCE are comparable to the ones that have been found previously, therefore confirming those isolated observations. Concerning reproduction processes, the spawning behavior of *P. noctiluca* has been widely studied^26,29,34,51^ showing that all sexually mature organisms spawned daily until their death. This gamete emission occurs 3 hours after the start of the light and for 30 minutes. However, this behavior could also be the result of laboratory conditions. Our new model parameterized with SMCE suggested that an almost daily spawning frequency is optimal for error minimization both in the laboratory and field conditions. Spawning activation was mostly discussed for the species *P. noctiluca*, but spawning among scyphozoans seems to be related to environmental stress such as light variations^34,52–55^ temperature^56^, and food availability^26,56^. Our model supposed that mucus production during spawning represented respectively 67.5%, 62.5%, and 67.5% of total spawn in the laboratory, *in situ*, and both conditions compared to literature, which estimated at 52%^34^. Our modeling results confirm that scyphozoan produced massive amounts of eggs daily to promote growth population and survival^57^.

To our knowledge, no experiment investigates the DOC excreted by *P. noctiluca*. Our simulations estimated an excretion rate close to 0 in all conditions. In comparison, two different studies measured excretion rates for *Mnemiopsis leidyi* (Ctenophora) ranging between 0.4 and 61.6 μmolC.gDW^−1^.h^−1 ^^58^ or 0.18 and 0.86 μmolC.gDW^−1^.h^−1 ^^59^ with temperature ranges of 18–27 °C and 10–24 °C, respectively. *Chrysaora quinquecirrha* and *Aurelia aurita* scyphomedusae, which are closer to *P. noctiluca* from a phylogenetic point of view, released high quantities of DOC with estimates spanning 1.3–46.4 μmolC.gDW^−1^.h^−1 ^^58^ and 1.2–6.7 μmolC.gDW^−1^.h^−1 ^^60^ in temperature ranges of 14–27 °C and 16–20 °C, respectively. Most of the time, jellyfish release a large amount of highly labile DOC, which can be easily metabolized by bacterioplankton with uptake rates two or six times that bulk of DOM^10^. Moreover, the authors showed that specific bacterial groups in the water column successfully used this matter and suggested that jellyfish promote fundamental transformation in the biogeochemical functioning and microbial loops.

In addition to rejecting specific parameter sets, we used the SMCE approach to evaluate the entire structure of models. The limits of the ecophysiological model used in this study were highlighted by the assimilation efficiency and excretion rate, which tend toward the extreme values (100% and 0% respectively) in both conditions simultaneously. Even if *a*_*max*_ values were unrealistic for *in situ* and both conditions simulations, it reached 80% in laboratory experiments. Moreover, the relationship between “Score *in situ*” and “Score growth” with *a*_*max*_ parameter had opposite correlations (Fig. 6), which means that to reduce simulation errors in the laboratory (*in situ*) condition, the assimilation efficiency values need to be lowest (highest). Here, the SMCE output analysis raises the common problem of modeling between parameterization in the field and controlled conditions.

Regarding the excretion rate, the SMCE results showed that the variability of size measurements during the degrowth experiments did not allow to constrain it, which was also confirmed by the correlogram where *c*_*e*_ does not affect laboratory score but seems to be the most influential parameters for the field conditions. To conclude, the correlogram showed that a good “Match” mostly depends on the growth in laboratory conditions, which itself largely depends on the laboratory prey concentration. These results suggest that, in addition to *c*_*e*_ and *a*_*max*_, the vector of the laboratory prey concentration tested and adapted following publication^42^ need to be estimated more precisely in the future experiments.

**Figure 7:**
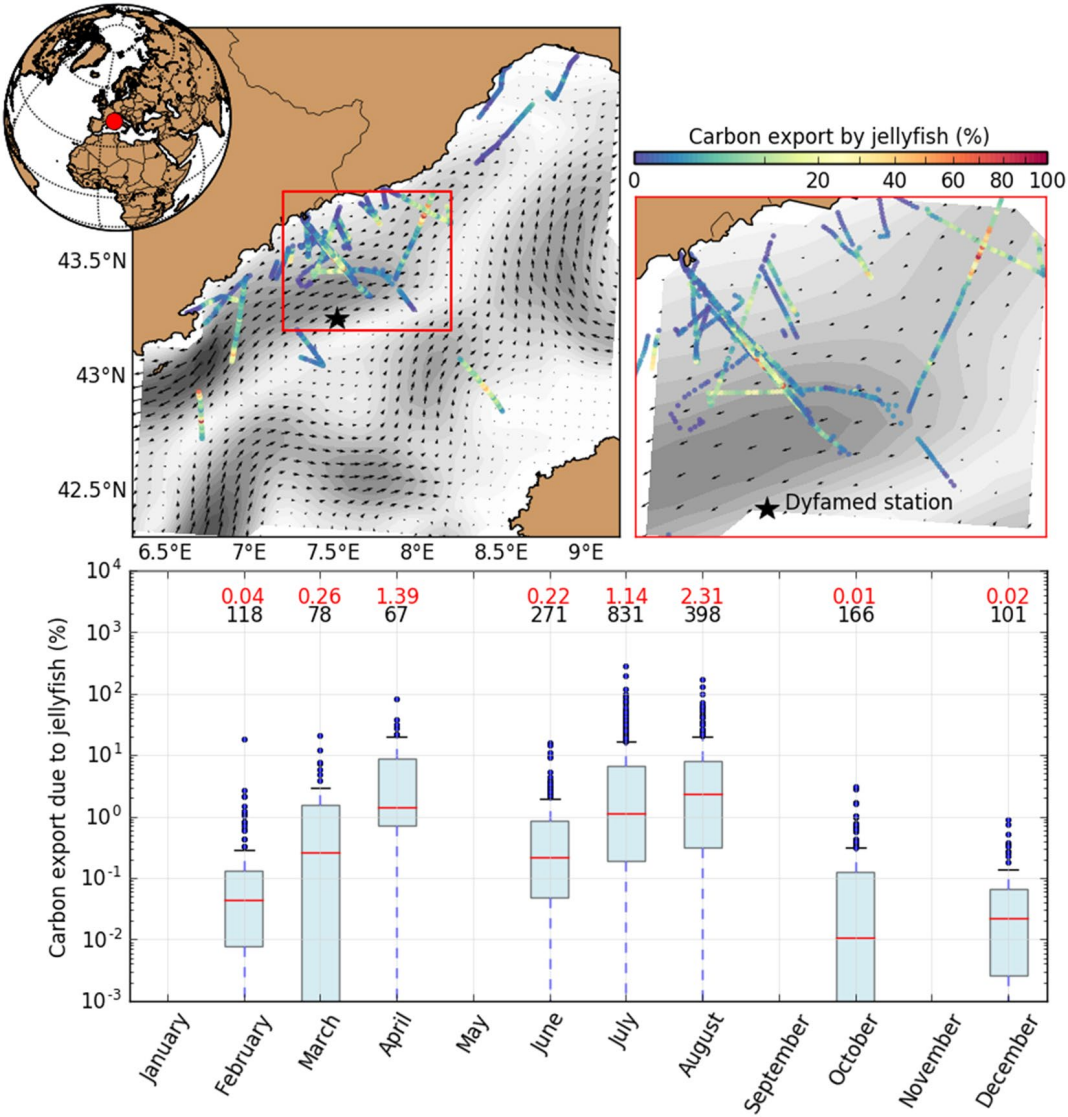
Spatiotemporal variability of the percentage of POC exported at 200 m depth due to the scyphozoan egestion in the Ligurian Sea, according to the ecophysiological model and compared to the total POC measured at the DYFAMED station in Ramondenc, *et al*.^61^. The red and black numbers represent the mean of POC percentage exported at 200 m depth and the number of observations respectively.

The present ecophysiological model provides carbon fluxes for predation, ingestion, assimilation, respiration, excretion, reproduction, and the egestion of *P. noctiluca*. Combining the modeled egestion with *P. noctiluca* abundances in the North-West Mediterranean Sea and estimated remineralization, and sinking rates of their produced mucus could enable estimating their contribution to carbon export in the region in the future (Eq. 9). For example, considering that in 2013, *P. noctiluca* abundances ranged from 0 to 3.45 ind.m^−2^ with a median of 0.018 ind.m^−2^ (1^st^ and 3^rd^ quartiles: 0.003 and 0.1 ind.m^−2^ respectively; *n* = 1,371). We could estimate an average mucus export at 200 m depth between 3.10^−3^ and 0.2 mgC.m^−2^.d^−1^ in October and April, respectively. In comparison with the total carbon export at DYFAMED station^61^ (ranging from 1.53 mgC.m^−2^.d^−1^ in July to 14.01 and 13.55 mgC.m^−2^.d^−1^ in February and April, respectively), this suggested that the contribution of *P. noctiluca* (%POC_jelly_) at 200 m in the region could range from 0.01% to 2.31% in October and August respectively, while in summer this contribution could vary between 0% and 288%, with a median value of 1.01% (first and third quartile: 0.15% and 5.6% respectively; Fig. 7). While this example shows the potential of the common use of the ecophysiological model and experiments to estimate the jellyfish contribution for carbon fluxes, it still major pitfalls that advocate for further modelings. The current model does not consider mortality and jelly falls, which could all impact fluxes estimates. Also, local POC_jelly_ predictions are compared to the monthly climatology of total POC fluxes obtained at the DYFAMED station^61^, which is the only time series available for this region. The Supplementary Information thoroughly discusses these hypotheses and potential impacts (section *Model uncertainties and putative refinements*).

This probabilistic modeling, initially designed as a determinist model, could suggest a weak impact of *P. noctiluca* on carbon fluxes, but the high biomass and prevalence of this species make it an excellent candidate for the jelly-POM concept^11^. Indeed, similarly to what has been found for Thaliacea^62–64^, the relatively high abundances and predation rates of scyphozoans can impact planktonic communities locally^65^, which therefore needs to be integrated into pelagic ecosystems modeling studies. Also, carcass decomposition coupled with excessive jellyfish excretion, can increase dissolved inorganic carbon concentration in the water column, and thus promotes the microbial food web^10^. Overall, this modeling exercise allows us to scale up from physiological properties, to test for potential biogeochemical implications, integrating laboratories, and *in situ* observations of the jellyfish *P. noctiluca*.

## Methods

### Conceptual model

To understand the role played by jellyfish for carbon cycling, it is essential to trace each physiological process where carbon is implicated. An ecophysiological 0-D model was built based on three forcing variables, which are temperature (T), food concentration (F) and initial jellyfish carbon mass (CM). The growth (G; gC.d^−1^) of any organism depends on the balance between the quantity of assimilated preys (A) and losses due to respiration (R; gC.d^−1^), excretion (Ex; gC.d^−1^) and reproduction (Re; gC.d^−1^). After ingestion (I; gC.d^−1^), unassimilated predated preys are considered as egestion (Eg; gC.d^−1^). In this way, the scope of growth and Eg could be expressed respectively by equations Eq. 1 and Eq. 2 below. Each variable was determined separately.

1 $$\varDelta G=A-(R+{Ex}+{Re})$$

2 $${Eg}=I-A$$

To estimate the assimilation rate, an estimation of ingested prey itself subject to the predation rate (P) and prey concentration (F) is necessary. The total amount of predated food is a function of an allometric relationship that depends on individual carbon mass (CM), temperature (T; °C) and food concentration, as follows:

3 $$P=1/(F+{k}_{p}).{p}_{{\max }}.{{CM}}^{{bp}}.{{t}_{10}}^{T}$$

where *p*_*max*_ is the maximum net feeding rate, *t*_10_ the tenth root of the *Q*_10_ which describes by how much rate changes with a 10 °C increase, and *b*_*p*_ is the regression coefficient. The parameters *p*_*max*_, *b*_*p*_, and *t*_*10p*_ were calibrated following the relationships between carbon weight and clearance rate defined for 8 different jellyfish species by Acuna, *et al*.^66^ (Fig. S1). Michaelis-Menten kinetics were performed in the predation equation, with a half constant saturation of feeding rate (*k*_*p*_). However, the parameters *p*_*max*_ and *k*_*p*_ are interconnected. To minimize the time needed for the new parameterization approach, we assumed that the most laboratory observations^66^ corresponds to food saturating condition (*i.e*., two times higher than the maximum food concentration recorded in the *in situ* condition; *F*_*sat*_ = 0.00012 gC.L^−1^) accordingly to which the relationship between *p*_*max*_ and *k*_*p*_ were identified, such as *p*_*max*_ = 0.1399.(1 + *k*_*p*_/*F*_*sat*_).

In this model, the ingestion rate is expressed by the simple product between prey concentration and predation rate, as follows:

4 $$I=P.F$$

However, the assimilation rate is different than the ingestion one and the model assumes that the fraction of food ingested and effectively assimilated decreases with increasing zooplankton concentration following a Holling type II Michaelis-Menten relationship:

5 $$A=I.(1-{a}_{{\max }}.F/(F+{k}_{a}))$$

where *a*_*max*_ is the maximum fraction of assimilated food and *k*_*a*_ is the half-saturation constant for assimilation.

To estimate the organic carbon losses due to the jellyfish metabolism, we considered four main processes: respiration, excretion, reproduction, and egestion rate. Many authors investigated jellyfish respiration rates^34,66^ but we choose to adapt the approach of Lilley, *et al*.^34^, which performed an allometric equation on *P. noctiluca* species such as:

6 $$R={R}_{o}.{{CM}}^{\mathrm{br}}.{{t}_{10}}^{T}$$

where *R*_*o*_ is the theoretical respiration rate for 1 g CM individual at 0 °C, *b*_*r*_ is the allometric exponent for the effect of individual mass on respiration rate and *t*_*10*_ is the tenth root of the *Q*_*10*_ coefficient. To convert oxygen to energy units and then energy units to carbon units, two parameters *α* (J. μmolO_2_^−1 ^^67^;) and *β* (gC.J^−1 ^^36^;), were used respectively. The parameters *R*_*o*_, *b*_*r*_, and *t*_*10r*_ were calibrated according to log-log relationship between carbon weight and respiration rate obtained by Lilley, *et al*.^34^ (Fig. S2).

Jellyfish excretion has been largely studied^10,58^. DOC concentration from excretion activity was considered to scale with respiration rates, which is representative of the overall metabolic rates, as follows:

7 $${Ex}=R.{c}_{e}$$

with c_e_ being the scalar factor.

Concerning the reproduction, when *P. noctiluca* is sexually mature, each individual spawning releases hundreds of eggs bound into mucus. In our model, eggs production was estimated thanks to the power-law relationship developed by Lilley, *et al*.^34^ (Fig. S3) and two additional parameters *c*_*re*_ and *spn*, that characterize mucus (as the proportion of eggs carbon) and spawning frequency (spawn per day) respectively following the equation Eq. 8:

8 $${Re}=({a}_{{re}}.{{CM}}^{{bre}}).{W}_{e}.{c}_{{re}}.{spn}$$

where *a*_*re*_ and b_re_ are coefficient parameters and *W*_*e*_ is the egg carbon mass^38^. Sexual maturity allowing egg production was attained when jellyfish size reached 4 cm^29^.

To estimate the contribution of POC_jelly_ to the carbon pump, the sinking speed (*w*; m.d^−1^) and the remineralization rate (*k; %*.d^−1^) were implemented in our model after estimation by laboratory experiments. The amount of carbon sequestered by jellyfish at a given depth was then modeled as follows:

9 $${C}_{{seq}}={Eg}.w\mathrm{.k}$$

In order to adapt the equations found in literature and switch easily between morphometric units, the model uses two conversions developed by Lilley, *et al*.^34^. Thus, the jellyfish carbon mass represents 0.36% of the wet mass. Moreover, the wet jellyfish mass and carbon mass follow an allometric law with the size of *P. noctiluca*, such as:

$$\mathrm{WM}=0.075.{\mathrm{BD}}^{2.993}\mathrm{and}\,\mathrm{CM}=0.26.{\mathrm{BD}}^{3.017}$$

with BD, WM, and CM representing respectively the bell diameter over lappets (*cm*), wet mass (*g*) and carbon mass (*mg*).

### Model execution and observation data

To correctly represent growth and degrowth of *P. noctiluca*, model outputs were compared to our laboratory and *in situ* measurements as well as those carried out by Lilley, *et al*.^38^. According to Lilley, *et al*.^34^, degrowth and growth experiments were carried out in a room maintained at 18 °C. Regarding food concentration, no prey was used in degrowth experiments whereas jellyfish were fed *ad libitum* during growth measurements. Facing important uncertainties that represent the term “*ad libitum”*, a food concentration vector specific to laboratory condition was tested with the SMCE approach (*F*_*lab*_, see Table 1).

An annual climatology based on weekly measurements of sea surface temperature and zooplankton concentrations, recorded from plankton imaging, were calculated for the 2011–2015 period. Zooplankton biomass was estimated in carbon units by converting zooplankton biovolume to biomass following the linear model of Alcaraz, *et al*.^68^, which was developed for the northwestern Mediterranean Sea. As previously mentioned, *P. noctiluca* undergoes nycthemeral migrations from the surface towards the bathypelagic^69^. This behavior was integrated by calculating ecophysiological rates for both sea surface temperature and bathypelagic temperature (*i.e*., 13 °C in the Mediterranean Sea). Then, the average of each ecophysiological rate at time *t* was computed to represent the average daily rates.

### SMCE processing

We ran 500 simulations for each parameter set which gave a 5% estimation precision and an error rate of 1% on the probability that the parameter set is correct (*i.e*., qualified as *Match*). The experiment was performed on a 64 cores CPU, 512 Go Ram, 13 To HDD computer, and ran for 8 080 minutes (*i.e*., 6 days). In the end, the SMCE returned all parameter sets that presented a *Match* greater than 70%. Additional characteristics were also returned, such as the average number of simulations that were found outside the confidence interval defined from data observation (*Score*), and the distance to the interval (*Dist*) together, for laboratory and *in situ* conditions. The ecophysiological model implementation is available at https://gitlab.univ-nantes.fr/delahaye-b/Pelagia-Noctiluca.

### Abundance and biomass estimation

The Ligurian Sea is an area in the northwestern Mediterranean Sea that presents its own cyclonic circulation^70^. Indeed, two different Modified Atlantic Waters (MAW) are mixed around Corsica and formed the “Northern current”. This strong flow progresses anticlockwise eastward along the Italian and French coasts. The Northern current exhibits specific physical and biological conditions and splits the basin into three different hydrographic zones: (*i*) the peripheral zone, (*ii*) the frontal zone and (*iii*) the offshore central zone. Our study monitored the distribution of *P. noctiluca* from 2012 to 2015^71^ with a quantitative method. Briefly, thanks to the French ship (www.alchimie-mediterranee.fr/, Alchimie), each cruise was carried out at night from Villefranche-sur-Mer (43°41′N, 7°18′E) to Calvi (42°34′N, 8°45′E). During this transect, only adult *P. noctiluca* were recognized and counted every 10 minutes. The jellyfish biomass was estimated thanks to the wet mass from sampled adult individuals.

### Sinking and remineralization rate experiments

Adult *P. noctiluca* were collected at the surface with a dip net (1 mm mesh size) close to the frontal zone. Each individual jellyfish was placed in an 8 L plastic bucket filled with filtered (100 μm) *in situ* seawater. Few hours were necessary for the gelatinous organisms to egest their gut content in the form of digestive mucus. The excreted particulate matter was individually incubated in sealed vials of 33.4, 67.9 or 136.5 mL filled with filtered (0.2 μm) seawater to determine the remineralization rate. Each vial contained small optodes foil glued inside their glass wall^72^. Optodes excited by light pulses emit a fluorescence pulse in response that depends on the oxygen concentration of the solution (quenching). The phase delay of the light response provides oxygen saturation rates for the incubator. This system (optical electrodes Presens ©), is a non-intrusive and precise method (precision: 0.4% of O_2_ air saturation) but it is sensitive to temperature and pressure. For this reason, it is necessary to calibrate each vial under controlled conditions before experimental measurements (need to re-calibrated every 2 months). In parallel, other vials were prepared without particles to obtain the control condition. Average respiration from control measurements was subtracted to experimental measurements with gelatinous mucus. All incubations (*n* = 33) ranged between 9–20 h. At the end of the experiment, each particle of mucus was frozen at −60 °C for CHN analysis. The degradation rate of organic matter was calculated thanks to a linear regression based on the decrease of O_2_ in time. Thanks to the final amount and degradation rate, both carbon content at the initial time and remineralized can be estimated. In another way, sinking speed rate experiments were conducted in the laboratory. The mucus remineralization rate was estimated at 0.034 d^−1^ function to the CO_2_ consumption measurements (Table S1).

All other individual digestive mucus was used to estimate particles sinking speed, inspired by the previous study^73^. One by one, each particle (*n* = 19) was placed in the first centimeters of a large graduated plastic bucket, which was 38 cm tall and 30 cm in diameter, full with filtered *in situ* seawater (0.2 μm) in order to conserve identical water density. The time taken by the particles to reach between 15 and 20 cm was used to estimate the sedimentation rate. The sinking rate of the mucus produced by *P. noctiluca* ranges between 384 m.d^−1^ and 1329 m.d^−1^ with a median equal to 751 m.d^*−*1^ (Table S2). The sinking rates were positively correlated with the mucus size (r^2^ = 0.6, *p* < *0.05*) and showed similar properties to marine snow. Finally, based on these two estimates, a mucus sinking between 0 and 200 m is expected to lose 1% of its carbon weight.

## Supplementary information

Supplementary information.
