# Supplementary material for: Probabilistic modeling to estimate jellyfish ecophysiological properties and size distributions
Source: Sci Rep. 2020 Apr 8;10:6074. doi: 10.1038/s41598-020-62357-5 (PMC7142072; doi:10.1038/s41598-020-62357-5)
Supplement: Supplementary file 1 — Supplementary information. [file 41598_2020_62357_MOESM1_ESM.pdf]

Supplementary Information

Probabilistic modeling to estimate jellyfish ecophysiological properties and size distributions

Simon Ramondenc[1*], Damien Eveillard[2], Lionel Guidi[1,3], Fabien Lombard[1], Benoît Delahaye[2]

[1] Sorbonne Université, CNRS, Laboratoire d'Océanographie de Villefranche, LOV, F-06230 Villefranche-sur-Mer, France

[2] Université de Nantes, CNRS, LS2N, F-44322 Nantes, France

[3] University of Hawaii, Department of Oceanography, Honolulu HI 96822, USA

[*]*Corresponding author: Simon.Ramondenc@obs-vlfr.fr*

Supplementary Text

Model uncertainties and putative refinements

The ecophysiological model relies on several hypotheses regarding parameter values and forcing variables. The parameter values are left unchanged throughout the entire life cycle, except for the reproduction rate, which was modeled once the jellyfish measure more than 4 cm. After fecundation, the eggs quickly change in shape from oocytes to adults passing by planula and juvenile stage. These morphological changes could significantly affect physiological processes. Nevertheless, all these modifications due to their growth were indirectly integrated by the allometric function body carbon mass variations in each equation.

A large uncertainty was assumed concerning the prey concentration input in the model. In controlled conditions, food concentration provided to the culture was considered constant whereas, in the original study[1], authors specified that jellyfish were fed *ad libitum* daily during growth experiment which supposes daily pulse of food concentration followed by a decrease while jellyfish consumes their prey. Moreover, in captivity jellyfish growth is limited by space contrary to *in situ* conditions, where food availability is the main limiting factor. Nevertheless, this inherent bias is mainly significant after the exponential stage, when growth reaches its equilibrium. Given that the SMCE was applied to observations obtained before the exponential stage, mistakes due to laboratory cultures were considered to be not significant and give us large confidence in our results. Moreover, as mentioned in the main text, the development of *P. noctiluca* is strongly controlled by environmental food and temperature, but this species also performs nycthemeral migrations from the surface to the deep ocean, so it actually experiences a gradient of these two factors. Regarding temperature, the gradient variation was considered towards the bathypelagic zone, but for the prey concentration, the model suggested that jellyfish are following them and therefore that the food abundance is constant from the surface to the deep ocean.
The mucus sinking speed experiments also present some uncertainties. The jellyfish egestion products take the shape of gelatinous strings. The particle sinking speed is mainly dependent on shape complexity[2]. However, to measure different sinking runs, samples were extracted from jellyfish tanks using a wide pipette to transfer it to the vertical-settling column. Consequently, the strings' shape could be modified towards a more compact shape. The present particles sinking speed could be overestimated, but this would not change its density which is comparable to similar types of marine snow. Similarly, variations of seawater temperature, salinity and density could modify remineralization and sinking rates[3,4]. Even if all experiments were performed with surface seawater at ambient temperature, the model supposed that both rates were constant for each season and depth. This error is nonetheless considered insignificant and conservative given the variability and standard deviation between measurements.

Supplementary Figure

Captions

Figure S1. Log-log plot of temperature-corrected clearance rates as a function of body carbon weight for several jellyfish species[5].

Figure S2. Log-log plot of temperature-corrected respiration rates as a function of body carbon weight for several jellyfish species[5,6].

Figure S3. Log-Log plot of egg production as a function of bell diameter over lappets in the laboratory and in situ condition (data published come from [1,7]).

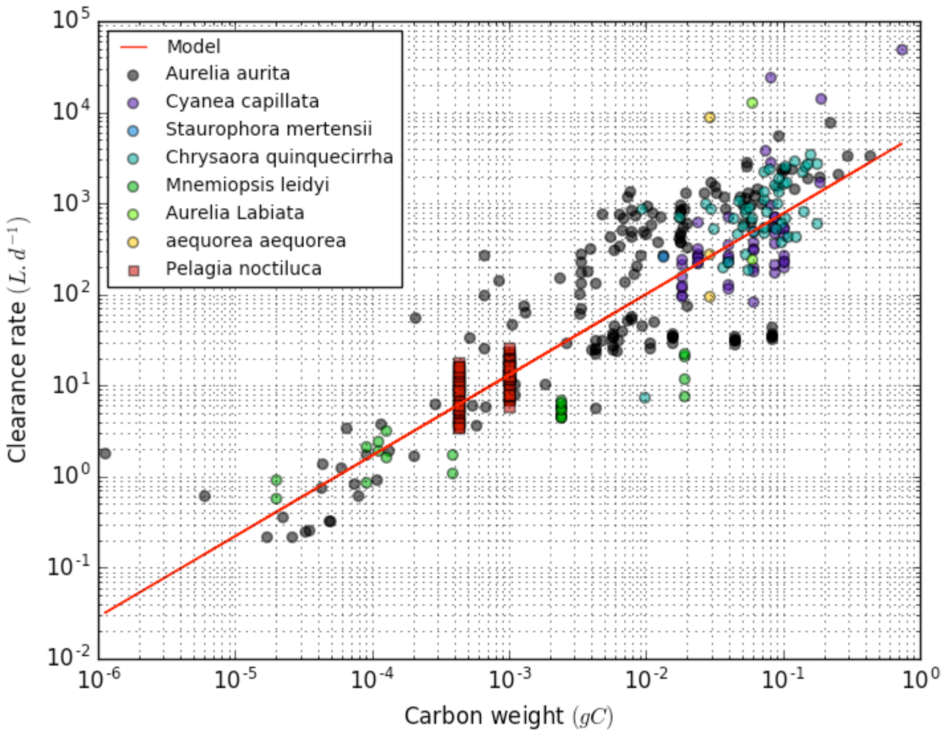

Figure S1

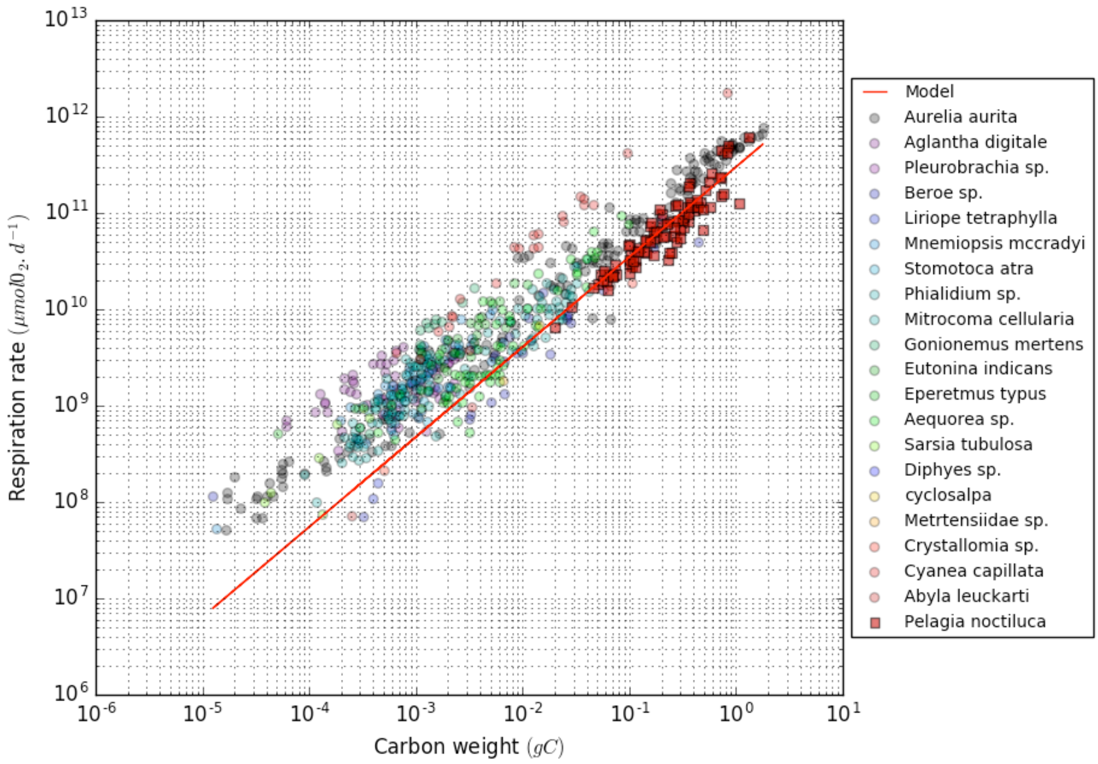

Figure S2

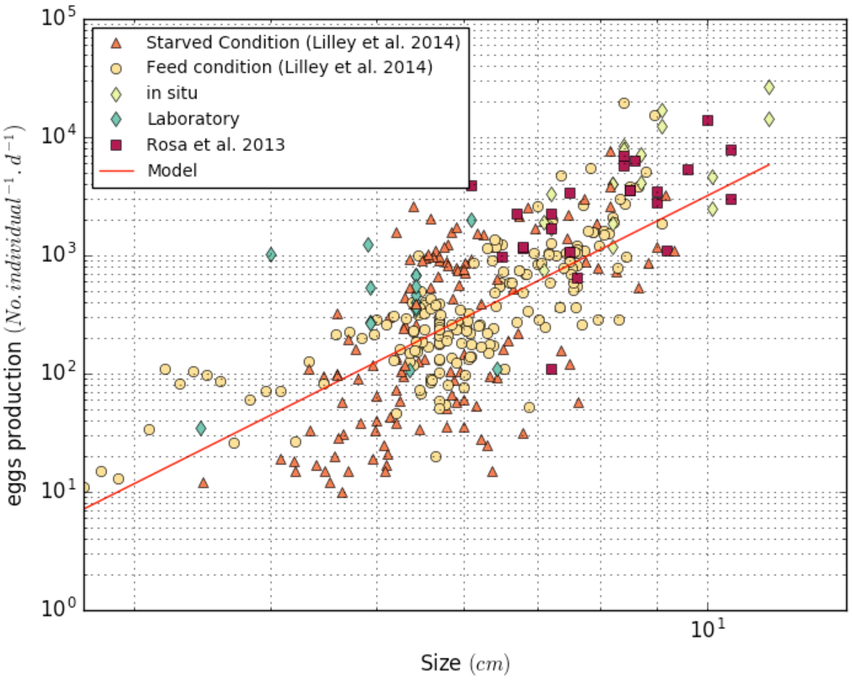

Figure S3

Supplementary Table

Captions

Table S1. Median with standard deviation of final mass of sampling, O_2 consumption, CO_2 production, matter remineralized and time estimated for total remineralization.

Table S2. Mean with a standard deviation of sinking time, distance traveled, surface, diameter spherical equivalent (DSE) and sinking speed measured in laboratory condition without turbulence of the particles O_2 consumption, CO_2 production, matter remineralized and time estimated for total remineralization.

Date	n	Final mass (mgC)	O_2 consumption (µmol.h^{-1})	CO_2 production (µmol.h^{-1})	Remineralized matter (%)	Remineralization time (d)
05/06/15	10	2.96 ± 1.54	0.29 ± 0.12	0.25 ± 0.10	3.40 ± 1.49	29.41 ± 29.05
24/06/15	7	2.93 ± 1.44	0.27 ± 0.30	0.24 ± 0.26	1.94 ± 1.76	51.63 ± 68.98
06/07/15	9	0.91 ± 0.95	0.30 ± 0.25	0.26 ± 0.22	8.96 ± 14.62	11.16 ± 18.89
09/10/15	4	0.3 ± 0.13	0.04 ± 0.02	0.04 ± 0.01	3.96 ± 4.09	26.75 ± 31.7
09/11/15	2	0.8 ± 0.11	0.09 ± 0.03	0.08 ± 0.02	2.79 ± 1.12	39 ± 15.68
10/11/15	4	0.44 ± 0.16	0.04 ± 0.02	0.03 ± 0.01	2.12 ± 0.21	47.29 ± 4.62

Table S1

Date	n	Time (s)	Distance (cm)	Surface (mm^2)	DSE (mm)	Sinking speed (m.d^{-1})	Carbon (%)	Sample mass (mg)
05/06/15	9	15 ± 6.54	15 ± 3.2	456.1 ± 199.4	24.1 ± 5.5	864 ± 262	1.22 ± 0.7	1.1 ± 1.2
06/07/15	10	27 ± 8.8	20 ± 0.0	585.2 ± 374	13.6 ± 5.0	640 ± 207	2.05 ± 1.01	3.5 ± 3.9

Table S2

References

1 Lilley, M. K. S. *et al.* Culture and growth of the jellyfish Pelagia noctiluca in the laboratory. *Mar Ecol Prog Ser* 510, 265-273, doi:10.3354/meps10854 (2014).

2 De La Rocha, C. L. & Passow, U. Factors influencing the sinking of POC and the efficiency of the biological carbon pump. *Deep-Sea Res Pt II* 54, 639-658, doi:Doi 10.1016/J.Dsr2.2007.01.004 (2007).

3 Lebrato, M. *et al.* Jelly biomass sinking speed reveals a fast carbon export mechanism. *Limnol Oceanogr* 58, 1113-1122, doi:10.4319/lo.2013.58.3.1113 (2013).

4 Tamburini, C. *et al.* Effects of hydrostatic pressure on microbial alteration of sinking fecal pellets. *Deep-Sea Res Pt II* 56, 1533-1546, doi:10.1016/j.dsr2.2008.12.035 (2009).

5 Acuna, J. L., Lopez-Urrutia, A. & Colin, S. Faking Giants: The Evolution of High Prey Clearance Rates in Jellyfishes. *Science* 333, 1627-1629, doi:10.1126/science.1205134 (2011).

6 Lilley, M. K. S. *et al.* Individual shrinking to enhance population survival: quantifying the reproductive and metabolic expenditures of a starving jellyfish, Pelagia noctiluca. *J Plankton Res* 36, 1585-1597, doi:10.1093/plankt/fbu079 (2014).

7 Rosa, S., Pansera, M., Granata, A. & Guglielmo, L. Interannual variability, growth, reproduction and feeding of Pelagia noctiluca (Cnidaria: Scyphozoa) in the Straits of Messina (Central Mediterranean Sea): Linkages with temperature and diet. *J Marine Syst* 111, 97-107, doi:10.1016/j.jmarsys.2012.10.001 (2013).
